# A Common Variant of *PNPLA3* (p.I148M) Is Not Associated with Alcoholic Chronic Pancreatitis

**DOI:** 10.1371/journal.pone.0029433

**Published:** 2012-01-20

**Authors:** Jonas Rosendahl, Anke Tönjes, Dorit Schleinitz, Peter Kovacs, Johannes Wiegand, Claudia Ruffert, Moritz Jesinghaus, Robert Schober, Max Herms, Robert Grützmann, Hans-Ulrich Schulz, Felix Stickel, Jens Werner, Peter Bugert, Matthias Blüher, Michael Stumvoll, Stephan Böhm, Thomas Berg, Henning Wittenburg, Joachim Mössner, Rene te Morsche, Monique Derikx, Volker Keim, Heiko Witt, Joost P. H. Drenth

**Affiliations:** 1 Division of Gastroenterology and Rheumatology, Department for Internal Medicine, Neurology and Dermatology, University of Leipzig, Leipzig, Germany; 2 Division of Endocrinology, Department for Internal Medicine, Neurology and Dermatology, University of Leipzig, Leipzig, Germany; 3 IFB Adiposity Diseases, University of Leipzig, Leipzig, Germany; 4 Interdisciplinary Center for Clinical Research Leipzig, University of Leipzig, Leipzig, Germany; 5 Department of General, Thoracic and Vascular Surgery, University Hospital Carl Gustav Carus, Technische Universität Dresden, Dresden, Germany; 6 Department of Surgery, Otto-von-Guericke University Magdeburg, Magdeburg, Germany; 7 Department of Visceral Surgery and Medicine, Inselspital, University of Bern, Bern, Switzerland; 8 Department of Surgery, University of Heidelberg, Heidelberg, Germany; 9 Medical Faculty Mannheim, Institute of Transfusion Medicine and Immunology, Heidelberg University, German Red Cross Blood Service of Baden-Württemberg-Hessen, Mannheim, Germany; 10 Department of Gastroenterology and Hepatology, Nijmegen Medical Center, Radboud University, Nijmegen, The Netherlands; 11 Department of Pediatrics and Else Kröner-Fresenius-Zentrum (EKFZ), Technical University Munich (TUM), Munich, Germany; Instituto de Ciencia de Materiales de Madrid - Instituto de Biomedicina de Valencia, Spain

## Abstract

**Background:**

Chronic pancreatitis (CP) is an inflammatory disease that in some patients leads to exocrine and endocrine dysfunction. In industrialized countries the most common aetiology is chronic alcohol abuse. Descriptions of associated genetic alterations in alcoholic CP are rare. However, a common *PNPLA3* variant (p.I148M) is associated with the development of alcoholic liver cirrhosis (ALC). Since, alcoholic CP and ALC share the same aetiology *PNPLA3* variant (p.I148M) possibly influences the development of alcoholic CP.

**Methods:**

Using melting curve analysis we genotyped the variant in 1510 patients with pancreatitis or liver disease (961 German and Dutch alcoholic CP patients, 414 German patients with idiopathic or hereditary CP, and 135 patients with ALC). In addition, we included in total 2781 healthy controls in the study.

**Results:**

The previously published overrepresentation of *GG*-genotype was replicated in our cohort of ALC (*p*-value <0.0001, OR 2.3, 95% CI 1.6–3.3). Distributions of genotype and allele frequencies of the p.I148M variant were comparable in patients with alcoholic CP, idiopathic and hereditary CP and in healthy controls.

**Conclusions:**

The absence of an association of *PNPLA3* p.I148M with alcoholic CP seems not to point to a common pathway in the development of alcoholic CP and alcoholic liver cirrhosis.

## Introduction

Chronic pancreatitis (CP) is a recurring or continuous inflammatory disease that can lead to permanent impairment of endocrine and exocrine function. In industrialized countries continued alcohol abuse is the most common underlying cause. Additionally, smoking increases the risk for CP [1]. Contrary to other alcohol-induced disorders, such as liver cirrhosis, only 5% of alcohol abusers develop CP [2]. In addition, familial clustering suggests a contribution of genetic alterations to the pathogenesis of alcoholic CP. In recent years several genetic associations have been discovered for hereditary and idiopathic CP [3]–[7]. Although, genetic case control studies in alcoholic CP have established robust associations with variants of serine protease inhibitor, kazal type 1 (*SPINK1*) and chymotrypsinogen C (*CTRC*) search for other risk variants have been less successful [8], [9]. However, a Japanese study performed a genome wide genetic analysis using microsatellite markers in alcoholic CP and discovered several susceptibility regions [10]. This suggests that whole genome information could provide new insights to understanding the pathogenesis of alcoholic CP and indicates that common variants might be of importance.

A genome wide association study in patients with non-alcoholic fatty liver disease revealed a link between *PNPLA3* (OMIM *609567) variants and liver fat content [11]. Subsequent investigations analysed *PNPLA3* variant p.I148M (rs738409) in alcoholic Mestizo individuals and demonstrated an overrepresentation of the *GG*-genotype in those with alcoholic liver disease [12]. This genetic association was confirmed in different cohorts from different ethnicities and the verdict is that rs738409 represents an independent risk factor for ALC with odds-ratios (OR) in the range of 2 that withstands multivariate analysis. In Caucasian alcoholics, rs738409 was also associated with elevated aminotransferase levels and in patients with chronic hepatitis C it appears to influence progression to fibrosis and development of steatosis [13]–[17].

Alcohol abuse is the predominant aetiology of CP and liver cirrhosis in industrialized countries. Given the fact that *PNPLA3* rs738409 is a risk factor for ALC it is evident to investigate its potential influence on the pathogenesis of alcoholic CP. In addition, both disease forms occur concomitantly in patients, albeit rarely, which might suggest a potential common pathway for disease development [18]–[21]. We therefore investigated *PNPLA3* variant rs738409 in patients with alcoholic CP and screened individuals with idiopathic and hereditary CP as well as a confirmatory cohort of ALC patients.

## Material and Methods

### Patients

The study protocol was approved by the medical ethical review committee of the University of Nijmegen (The Netherlands) and Leipzig (Germany) and all patients gave written informed consent. Diagnosis of CP was based on two or more of the following findings. Presence of a typical history of recurrent pancreatitis or recurrent abdominal pain typical for CP, pancreatic calcifications and/or pancreatic ductal irregularities revealed by endoscopic retrograde pancreaticography or by magnetic resonance imaging of the pancreas and/or pathological sonographic findings. Alcoholic CP was defined in patients who had consumed more than 80 g/d alcohol for at least two years in men and more than 60 g/d for women. Hereditary CP was diagnosed when one first-degree relative or two or more second-degree relatives suffered from recurrent acute or CP without any apparent precipitating factor. Idiopathic CP was diagnosed in the absence of a positive family history or possible precipitating factors.

ALC was diagnosed according to results of liver biopsy (fibrosis stage 4) or due to unequivocal clinical and laboratory findings in men who consumed more than 80 g/d and in women who consumed more than 60 g/d for at least 10 years. Such findings were abnormal levels of aminotransferases, gamma glutamyl transpeptidase, coagulation tests, serum albumin concentration, platelet count, complications related to liver cirrhosis like esophageal varices, ascites, hepatic encephalopathy and typical liver morphology in sonography or computed tomography. Other etiologies of liver cirrhosis were excluded by standard laboratory tests.

Overall, we enrolled 755 German patients with alcoholic CP (650 male, age range 23–82 years, median 48 years), 333 patients with idiopathic CP and 81 patients with hereditary CP (202 male, age range 1–75 years, median 20 years; known variants: *PRSS1* variants: p.R122H n = 16, p.N29I n = 5, p.A16V n = 1, p.D22G n = 1, p.L104P n = 1, p.R122C n = 1, p.C139F n = 1; *SPINK1* variants: p.N34S homozygous n = 11, p.N34S heterozygous n = 93, p.L14P n = 1) and 128 patients with alcoholic liver cirrhosis (97 male, age range 32–78 years, median 55.5 years). In addition, we investigated 206 Dutch patients with alcoholic CP (152 male, age range: 33–76 years, median 51 years) and 7 patients with ALC (6 male, age range 49–61 years, median 56 years). We analysed patients with alcoholic liver cirrhosis as one cohort. In total, we screened 1950 blood donors from south-western and eastern Germany (853 male, age range 18–81 years, median 49 years) and 831 healthy controls without CP from the Netherlands (377 male, age range 18–96 years, median 50 years).

### Genotyping

We extracted genomic DNA from peripheral blood leukocytes according to a standard protocol (Qiagen, Hilden, Germany) and performed polymerase chain reaction (PCR) using 0.75 U AmpliTaq Gold polymerase (Applied Biosystems, Inc. Foster City, CA, USA), 400 µM dNTPs, 1.5 mM MgCl_2_ and 0.4 µM F-Primer as well as 0.1 µM R-primer in a total volume of 25 µl. Cycle conditions were an initial denaturation for 12 minutes at 95°C followed by 35 cycles of 20 seconds denaturation at 95°C, 40 seconds annealing at 60°C, 90 seconds primer extension at 72°C and a final extension for 2 minutes at 72°C in an automated thermal cycler (Applied Biosystems, Carlsbad, USA). Primers were synthesized according to the published nucleotide sequences (*PNPLA3*: GenBank: NM_025225.2): F-Primer 5′-CTTCTCTCTCCTTTgCTTTCAC-3′, R-Primer 5′-gCAggAgATgTgTgAgCAC-3′.

We performed melting curve analysis in the LightCycler 480 instrument (Roche Diagnostics, Mannheim, Germany) using a pair of fluorescent resonance energy transfer (FRET) probes. FRET probes were designed complementary to the mutated sequence. Probes were devised and synthesised by TIB Molbiol (Berlin, Germany): Anchor probe 5′-ACCACgCCTCTgAAggAAggAgggATAAg-FL-3′, Sensor probe 5′-610-CCACTgTAgAACggCATgAAgC-3′. Analytical melting included the following steps: 95°C for 60 seconds, 35°C for 60 seconds and an increase to 70°C at a 0.14°C/s ramp rate. If melting curves distinguished differently than expected from WT or mutated samples, we performed DNA sequencing of the accordant sample to identify the responsible variant.

### Statistics

Power analysis was performed using QUANTO 1.1 in a case control model [22]. We used the detected allele frequency of the *G*-allele in German controls (23%) and performed analysis in an additive model. The corresponding samples to controls ratio was 2.6, respectively (German patients, n = 755; German controls, n = 1950).

We tested the significance of the differences between variant frequencies in affected individuals and controls by two-tailed Fisher's Exact test and calculated *p*-values using GraphPad Prism (v 4.03). For *PNPLA3* variant p.I148M we utilised a dominant model, defined as CC vs. GC+GG, for calculations and considered *p*-values <0.05 to be of statistical significance. In addition, calculations were performed following a recessive model (CC+GC vs. GG) and for allele frequencies.

## Results

### Power calculations

Considering a minor allele frequency of 23% that was obtained in German controls we had a statistical power of at least 0.9 (α = 0.05) to detect genetic effects with an OR ≥1.25. Of course effects below a threshold of 1.25 (OR) might be missed, but we reasoned that effects below this threshold should be of neglible relevance for the pathogenesis of ACP.

### *PNPLA3* and pancreatitis

In our cohort of 755 German patients with alcoholic CP we detected the rs738409 *GG*-genotype in 37/755 patients (4.9%) and found similar results in the Dutch group of patients with alcoholic CP (8/206, 3.9%) (Table 1). Upon comparison with German and Dutch controls our genotype data yielded similar distributions (dominant model, *p*-value 0.07, 0.7). This was similar for the overall cohort of alcoholic CP patients compared to the pooled controls (dominant model, *p*-value 0.2). Allele frequencies of the *G*-Allele were comparable in all groups (German ACP 323/1510, 21.4%; Dutch ACP 92/412, 22.3%; German controls 912/3900, 23.4%; Dutch controls 382/1662, 23%). However, when we summarized all patients with alcoholic CP and compared genotype and allele data with ALC patients we obtained a significant attributed risk. We found a *GG*-Genotype in 45/961 (4.7%) of all patients with alcoholic CP, compared to 25/135 (18.5%) of ALC patients (dominant model, *p*-value <0.0001, OR 2.3, 95% CI 1.6–3.3; 3.9-fold accumulation). If we focus on allele frequencies we found that the *G*-allele was detectable in 415/1922 (21.6%) alleles in all alcoholic CP patients, while in ALC the *G*-allele was present in 40.4% of all alleles (*p*-value <0.0001, OR 2.5, 95% CI 1.9–3.2).

**Table 1:** Genotype data of *PNPLA3* variant rs738409 in German and Dutch patients with alcoholic CP and in German and Dutch controls.

Variant	Genotype	Patients ACP	Controls	p-Value	OR
		German			
p.I148M c.444C>G rs738409	CC	469/755 (62.1%)	1135/1950 (58.2%)	n.s.	-
	GC	249/755 (33%)	718/1950 (36.8%)		
	GG	37/755 (4.9%)	97/1950 (5.0%)		
		**Dutch**			
	CC	122/206 (59.2%)	505/831 (60.8%)	n.s.	-
	GC	76/206 (36.9%)	270/831 (32.5%)		
	GG	8/206 (3.9%)	56/831 (6.7%)		
		**Pooled (German/Dutch)**			
	CC	591/961 (61.5%)	1640/2781 (59%)	n.s.	-
	GC	325/961 (33.8%)	988/2781 (35.5%)		
	GG	45/961 (4.7%)	153/2781 (5.5%)		

Percentages are given in brackets. *P*-values were calculated for these groups in a dominant (CC vs. GC+GG) and recessive model (CC+GC vs. GG). Abbreviations: ACP = alcoholic CP, n.s. = not significant.

In patients with idiopathic and hereditary CP allele frequencies of rs738409 *G*-allele (193/828, 23.3%; *p*-value 0.4) were comparable to those in controls, but the *GG*-genotype was more frequent in patients (31/414, 7.5%) than in controls (97/1950, 5.0%). There was also no statistical significant difference between groups with respect to genotype data (dominant model, *p*-value 1.0; recessive model, *p*-value 0.05).

### *PNPLA3* and alcoholic liver disease

In patients with ALC *GG*-genotype of rs738409 was more frequent (3.7-fold) and present in 25/135 (18.5%) patients compared to 97/1950 (5.0%) in German controls (dominant model, *p*-value <0.0001, OR 2.7, 95% CI 1.9–3.9) (Table 2). When we pooled all controls *GG*-Genotype was 3.4-fold more frequent, respectively (dominant model, 153/2781, 5.5%; *p*-value <0.0001, OR 2.4, 95% CI 1.7–3.4). In patients with ALC *G*-Allele accounted for 40.4% (109/270) of all alleles in comparison to 23.3% in controls (1294/5562; *p*-value <0.0001, OR 2.2, 95% CI 1.7–2.9).

**Table 2:** Genotype data of *PNPLA3* variant rs738409 in German (n = 128) and Dutch (n = 7) patients with alcoholic liver cirrhosis, German patients with idiopathic and hereditary CP and in German controls.

Variant	Genotype	Patients	Controls	p-Value	OR
		ALC	German		
p.I148M c.444C>G rs738409	CC	51/135 (37.8%)	1135/1950 (58.2%)	<0.0001	2.3 (1.6–3.3)
	GC	59/135 (43.7%)	718/1950 (36.8%)		
	GG	25/135 (18.5%)	97/1950 (5.0%)		
		**ICP/HP**	**German**		
	CC	240/414 (58%)	1135/1950 (58.2%)	n.s.	-
	GC	143/414 (34.5%)	718/1950 (36.8%)		
	GG	31/414 (7.5%)	97/1950 (5.0%)		

Percentages are given in brackets. *P*-values were calculated for these groups in a dominant and recessive model. The *p*-value represents the result of the dominant model (CC vs. GC+CC). Abbreviations: ALC = alcoholic liver cirrhosis, ICP = idiopathic CP, HCP = hereditary CP, n.s. = not significant.

## Discussion

The current study examines the influence of *PNPLA3* variant p.I148M in a large cohort of patients with alcoholic liver and pancreas disease and controls.

We confirmed the association of *PNPLA3 GG*-genotype with ALC in our cohort (*p*-value <0.0001) and the overrepresentation of *GG*-genotype compared to controls was 3.7-fold (OR 2.3, 95% CI 1.6–3.3), respectively 3.4-fold (OR 2.4, 95% CI 1.7–3.4) for the pooled control group, which is higher than found in recent studies [13], [14]. The difference might be due to a type 2 error as our ALC patient group is relatively small precluding an accurate estimation of the risk. Our analysis further supports the current knowledge that *PNPLA3* rs738409 represents a risk factor for ALC in different ethnic groups with odds ratios around two.

Apart from putative genetic associations, little is known on the functional consequences of *PNPLA3* variant p.I148M. Recent data demonstrated a reduced capacity of mutated PNPLA3 to hydrolyse triglycerides with subsequent accumulation in the liver [23]. One might speculate that also in patients with ACP triglyceride haemostasis is disturbed with the result of CP due to inflammatory processes in the pancreas. Due to the limited knowledge of PNPLA3 function available so far one has to be aware of the speculative character of the aforementioned theory.

Contrary to ALC, little is known about potential genetic associations of alcoholic CP, despite knowledge of the role of *SPINK1* and *CTRC* variants in CP. Some patients with alcoholic CP also suffer from ALC. Epidemiological data on simultaneous presence of ALC and ACP are scarce and according to recent findings more than one third of patients with alcoholic CP may have concomitant ALC while around 20% of patients with ALC suffer from CP [21]. It is worthy to point out that prevalence data on the coexisting of alcoholic CP and ALC are inconsistent among different studies [18]–[21]. The fact that precursor lesions of both disease forms, steatosis and pancreatic fibrosis, are often found concurrently, portends to potential concerted risk factors.

Our analysis of *PNPLA3* variant p.I148M in German and Dutch patients with alcoholic CP demonstrated similar data for genotype and allele frequencies, when compared to pooled controls (*p*-value 0.2 and 0.1). We also confirmed the association of *PNPLA3 GG*-genotype with ALC when we analysed patients with alcoholic CP and compared them to ALC (*p*-value <0.0001). As a consequence, the obtained data of *PNPLA3* variant rs738409 implicate that different pathways might be implicated in the development of alcoholic CP and ALC.

We screened patients with idiopathic and hereditary CP to describe the frequency of this variant in other forms of CP and found an overrepresentation of *GG*-genotype compared to our control group. However, calculation of genotype distribution in a dominant and recessive model (*p*-value 1.0 and 0.05) and comparison of allele frequencies (*p*-value 0.4) yielded no statistical significant different results. This genetic result accords with the commonly accepted pathophysiological model of idiopathic and hereditary CP.

### Study limitations

Our study comes with limitations. Part of the control group used for comparison are blood donors and as such this introduces potential bias since ALC or APC may be prevalent among blood donors. We surmise that the contribution of such an effect is rather small. Blood donors are routinely screened for elevated liver enzymes and patients with chronic alcohol abuse should not be suitable to donate blood. Of note, in blood donors there might be a selection of a distinct group (healthy persons that are willing to donate blood), which may affect the obtained results. However, according to the low incidence of both disease entities in the general population we do not believe that will alter the results substantially. We think that the risk for bias is limited because genotype frequencies of rs738409 in our controls are similar to that in other Caucasian controls reported so far [24], [25].

Most of all, it has to be noted that we only investigated one particular variant that was previously proven to be of importance for alcohol related liver disease. Our results seem to indicate, that the absence of an association of *PNPLA3* variant p.I148M with alcoholic CP does not point to a common pathway in the development of alcoholic CP and ALC. However, we are aware that our conclusions are limited by the fact that only one variant with a modest risk for ALC whose pathogenic role is largely unknown, was investigated in patients with ACP.
